# Improvement of Cardiac Function After Roux-en-Y Gastric Bypass in Morbidly Obese Patients Without Cardiac History Measured by Cardiac MRI

**DOI:** 10.1007/s11695-020-04543-y

**Published:** 2020-03-20

**Authors:** Dennis de Witte, Leontine H. Wijngaarden, Vera A. A. van Houten, Marinus A. van den Dorpel, Tobias A. Bruning, Erwin van der Harst, René A. Klaassen, Roelf A. Niezen

**Affiliations:** 1 Department of Radiology, Maasstad Hospital, Rotterdam, the Netherlands; 2 Department of Radiology, Erasmus Medical Centre, Rotterdam, the Netherlands; 3 Department of Surgery, Maasstad Hospital, Rotterdam, the Netherlands; 4 Department of Surgery, Deventer Hospital, Deventer, the Netherlands; 5 Department of Internal Medicine, Maasstad Hospital, Rotterdam, the Netherlands; 6 Department of Cardiology, Maasstad Hospital, Rotterdam, the Netherlands

**Keywords:** Obesity, Bariatric surgery, Roux-en-Y gastric bypass, Cardiac magnetic resonance imaging, Left ventricular ejection fraction, Left ventricular mass

## Abstract

**Purpose:**

Metabolic syndrome in patients with morbid obesity causes a higher cardiovascular morbidity, eventually leading to left ventricular hypertrophy and decreased left ventricular ejection fraction (LVEF). Roux-en-Y gastric bypass (RYGB) is considered the gold standard modality for treatment of morbid obesity and might even lead to improved cardiac function. Our objective is to investigate whether cardiac function in patients with morbid obesity improves after RYGB.

**Materials and Methods:**

In this single center pilot study, 15 patients with an uneventful cardiac history who underwent RYGB were included from May 2015 to March 2016. Cardiac function was measured by cardiac magnetic resonance imaging (CMRI), performed preoperatively and 3, 6, and 12 months postoperative. LVEF and myocardial mass and cardiac output were measured.

**Results:**

A total of 13 patients without decreased LVEF preoperative completed follow-up (mean age 37, 48.0 ± 8.8). There was a significant decrease of cardiac output 12 months postoperative (8.3 ± 1.8 preoperative vs. 6.8 ± 1.8 after 12 months, *P* = 0.001). Average myocardial mass declined by 15.2% (*P* < 0.001). After correction for body surface area (BSA), this appeared to be non-significant (*P* = 0.36). There was a significant improvement of LVEF/BSA at 6 and 12 months postoperative (26.2 ± 4.1 preoperative vs. 28.4 ± 3.4 and 29.2 ± 3.6 respectively, both *P* = 0.002). Additionally, there was a significant improvement of stroke volume/BSA 12 months after surgery (45.8 ± 8.0 vs. 51.9 ± 10.7, *P* = 0.033).

**Conclusion:**

RYGB in patients with morbid obesity with uneventful history of cardiac disease leads to improvement of cardiac function.

## Background

Morbid obesity is characterized by multiple pathophysiological processes leading to changes in metabolism and eventually functional impairment [1]. One of the obesity-related comorbidities is cardiac morbidity, including left ventricular hypertrophy (LVH) as well as diminished left ventricular ejection fraction (LVEF) [2]. Bariatric surgery is a well-established and effective treatment for morbid obesity, including improvement of obesity-related comorbidities such as hypertension, dyslipidemia, and type 2 diabetes mellitus [3]. Several studies have been performed to analyze changes in cardiac function in patients with preoperative cardiomyopathy, showing improvement in cardiac function after Roux-en-Y gastric bypass (RYGB) [4–6]. As measured by cardiac ultrasound (CUS), it has been stated that cardiac function may also benefit from bariatric surgery in patients without cardiac history, eventually resulting in improved left ventricular function (LVF) and diminished left ventriclemass (LVM) and diameter. This potentially leads to a decrease of LVH and an increase of LVEF [4, 7–9]. The decrease in body mass index (BMI) after bariatric surgery seems to be correlated with the decrease in LVM [2]. Cardiac magnetic resonance imaging (CMRI) is less seriously influenced by subcutaneous fat than CUS, and can determine functional parameters, such as dimensions of the left ventricle (LV), LVM, and LVEF [7, 10–13]. Therefore, the gold standard for measurement of cardiac function in patients with morbid obesity should be CMRI. Previous studies to assess changes in cardiac function in patients without a history of cardiac disease have been performed using CUS, and therefore we performed a pilot study to investigate whether cardiac function in patients with morbid obesity with uneventful cardiac history improves after RYGB as measured by CMRI [14–16].

## Methods

### Study Population

A total of 15 patients who underwent RYGB at the Maasstad Hospital in Rotterdam from September 2015 to May 2016 were included in this study. CMRI could not be performed in two patients as they were claustrophobic. Therefore, the data of these patients were excluded from this study.

### Surgical Procedure

All procedures were performed by experienced bariatric surgeons. First, a gastric pouch of 25 cc was created. A 50-cm biliopancreatic limb was measured and the gastrojejunostomy was created using an endostapler and a continuous, absorbable suture. A side-to-side jejunojejunostomy was created using an endostapler and a continuous, absorbable suture, with an alimentary limb of 150 cm. Afterward, a transection between both anastomoses of the jejunum was performed.

### Postoperative Care

Postoperative care was performed by our standard postoperative care protocol. In this protocol, all patients were seen at the outpatient clinic 2 weeks, 3, 6, 9, and 12 months postoperative. All patients were counseled by a dietician, consisting of two group sessions and four individual consultations in the first year postoperative. All patients were advised to consume a calorie-restricted, high-protein diet consisting of approximately 1000 cal per day and 60–80 g of protein per day. All patients were advised to do moderate-intensity physical activities for at least 30 min per day. In addition, patients were advised to exercise for 1 h at least twice a week.

### Cardiac Magnetic Resonance Imaging

Imaging was performed with a 1.5 Tesla Siemens Somatom Definition scanner (Siemens AG, Erlangen, Germany). Short axis multislice cine TRUE FISP series of the heart were obtained for LV function analysis. In addition, post contrast series using gadolinium contrast agent (Bayer AG, Leverkusen, Germany) were obtained for the detection of late enhancement, a parameter to objectify ischemic changes of the myocardium.

### CMRI Data Analysis

CMRI data analysis was performed using Siemens syngo.via versions 10 and 11 (Siemens AG, Erlangen, Germany). By drawing the endocardial and epicardial contours of the myocardium, a 3D model was obtained. Via this model, the LVEF, end diastolic volume (EDV), end systolic volume (ESV), stroke volume (SV), cardiac index (CI), myocardial mass (MM; at end diastolic phase), peak ejection rate (PER), and peak filling rate (PFR) were calculated.

In addition to functional CMRI studies, we obtained blood samples to evaluate the effects of RYGB on the metabolic syndrome in these patients, such as kidney function, liver function, and lipid spectrum. We also determined leptin and ghrelin and the cardiac NT-pro BNP and vWF antigen.

### Statistical Analysis

Body surface area (BSA) was measured using the Du Bois formula [17]:

$$ \mathrm{BSA}=0.007184\times \mathrm{Weight}\ {\left(\mathrm{in}\kern0.33em \mathrm{kg}\right)}^{0.425}\times \mathrm{Height}\ {\left(\mathrm{in}\kern0.33em \mathrm{cm}\right)}^{0.725} $$

Statistical analysis was performed with IBM SPSS Statistics, version 23 (SPSS, Chicago, IL). Continuous data are presented as the mean ± standard deviation. Percentage excess weight loss (%EWL) was measured with the ideal weight defined by the weight corresponding to a BMI of 25 kg/m^2^. Analysis of repeated measures was performed using linear mixed models. A *P* value < 0.05 was considered significant. Missing data were addressed with pairwise deletion of missing data.

## Results

### Clinical Characteristics

A total of 13 patients with a mean age of 48.0 ± 8.8 years were included in this study, of whom 8 (61.5%) were female. There was a significant increase of %EWL of 46.4%, 67.8%, and 84.5% % at 3, 6, and 12 months after surgery respectively (*P* < 0.001). As a result, BSA decreased significantly after 3, 6, and 12 months, from 2.3 m^2^ preoperative to 2.0 m^2^ after 12 months. Heart rate and systolic blood pressure decreased significantly after 6 and 12 months (*P* < 0.001) (Table 1).

**Table 1:** Clinical characteristics

Variable	Preoperative	Postoperative (months)		
		3	6	12
Pulse (/min)	83.8 ± 16.2	72.3 ± 12.8	67.5 ± 9.5*	66.3 ± 12.2*
Systolic BP	143.3 ± 22.6	126.3 ± 10.5*	127.2 ± 11.9*	133.1 ± 22.7*
Diastolic BP	86.6 ± 14.3	87.9 ± 13.2	88.9 ± 10.1	89.4 ± 14.8
BMI (kg/m^2^)	40.1 ± 2.1	33.2 ± 2.6*	30.0 ± 2.7*	27.5 ± 3.8*
BSA	2.3 ± 0.2	2.1 ± 0.2*	2.1 ± 0.1*	2.0 ± 0.2*
%TWL		17.0 ± 4.2	25.1 ± 5.5†	31.2 ± 8.1
%EWL		46.4 ± 14.0	67.8 ± 17.2†	84.5 ± 23.2†

*BP*, blood pressure; *BMI*, body mass index; *BSA*, body surface area; *%TWL*, percentage total weight loss; *%EWL*, percentage excess weight loss

*Significant (*P* < 0.05) versus preoperatively

†Significant as compared to %TWL or %EWL after 3 months using the paired Student’s *T* test

### Changes in Cardiac Function

**Fig. 1:**
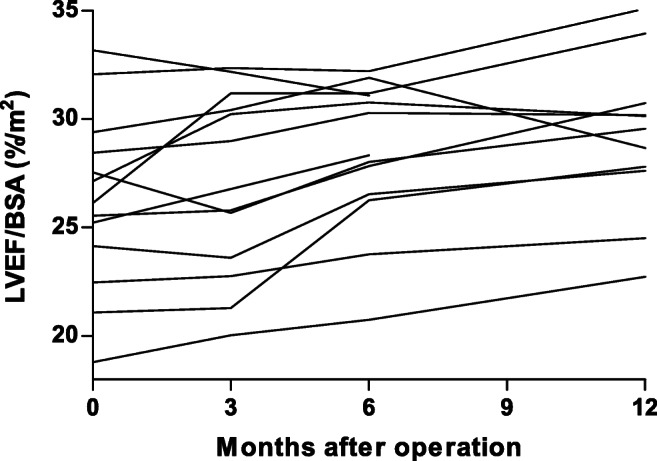
Changes in left ventricular ejection fraction / body surface area ratio. LVEF, left ventricular ejection fraction; BSA, body surface area

Cardiac output declined significantly 12 months after bariatric surgery (8.3 ± 1.8 vs. 6.8 ± 1.8, *P* = 0.001). Heart rate declined significantly at 6 and 12 months after bariatric surgery (67.5 ± 9.5 and 66.3 ± 12.2, *P* < 0.05). The average MM declined by 15.2% (*P* < 0.001) (Table 2). However, after correction for changes in BSA, no significant decline was seen 12 months postoperative (*P* = 0.36). LVEF declined significantly only at 3 months postoperative (56.6 ± 6.6, *P* < 0.05). However, after correction for BSA, there was a significant increase in LVEF/BSA ratio 6 and 12 months postoperative (both, *P* = 0.002) (Fig. 1). SV did nog change significantly. Additionally, there was a significant increase in SV/BSA ratio after 12 months follow-up (45.8 ± 8.0 versus 51.9 ± 10.7, *P* = 0.033). These results did not change after exclusion of one female patient in whom a right bundle branch block was found by coincidence. No patients had delayed myocardial enhancement. Detected by CMRI, all patients had hepatic steatosis preoperative, which completely disappeared 3 to 6 months postoperative in all study subjects.

**Table 2:** Cardiac function based on magnetic resonance imaging

Variable	Preoperative	Postoperative (months)		
		3	6	12
MRI				
LVEF (%)	60.2 ± 6.9	56.6 ± 6.6*	58.3 ± 6.1	58.0 ± 6.3
ED volume (ml)	177.6 ± 35.0	176.8 ± 44.9	183.5 ± 46.3	180.1 ± 43.6
ES volume (ml)	71.3 ± 20.1	77.2 ± 24.0	76.8 ± 22.5	71.3 ± 20.1
Stroke volume (ml)	106.3 ± 22.2	99.5 ± 25.9	106.7 ± 28.1	104.8 ± 29.7
Cardiac output (l/min)	8.3 ± 1.8	6.7 ± 1.3*	6.6 ± 1.4*	6.8 ± 1.8*
Myocardial mass (ED, in g)	127.4 ± 35.5	113.2 ± 33.3*	111.7 ± 31.1*	111.8 ± 34.0*
Myocardial mass (Average, in g)	140.6 ± 35.7	121.2 ± 34.3*	115.8 ± 30.6*	119.2 ± 36.0*
Peak ejection rate (ml/s)	− 505.7 ± 135.3	− 479.5 ± 93.7	− 471.0 ± 92.6	− 471.3 ± 116.2*
Peak ejection time (ms)	113.5 ± 20.8	125.3 ± 34.9	131.9 ± 26.0*	146.6 ± 28.3*
Peak filling rate (ml/s)	501.5 ± 78.8	447.0 ± 116.6	438.3 ± 143.7*	443.8 ± 113.5*
Peak filling time (ms)	546.5 ± 101.5	660.4 ± 198.9	527.0 ± 209.2	622.0 ± 180.1
MRI/BSA				
LVEF/BSA (%/m^2^)	26.2 ± 4.1	27.0 ± 4.4	28.4 ± 3.4*	29.2 ± 3.6*
ED volume/BSA (ml/m^2^)	76.5 ± 12.0	83.0 ± 16.5*	88.4 ± 18.0*	89.3 ± 15.2*
ES volume/BSA (ml/m^2^)	30.7 ± 8.0	36.1 ± 9.1*	37.1 ± 9.6*	37.4 ± 7.6*
Stroke volume/BSA (ml/m^2^)	45.8 ± 8.0	46.9 ± 10.5	51.4 ± 11.1*	51.9 ± 10.7*
Cardiac index (ml/min/m^2^)	3.6 ± 0.6	3.1 ± 0.5*	3.2 ± 0.6*	3.4 ± 0.7
Myocardial mass/BSA (ED, in g/m^2^)	54.4 ± 11.1	52.9 ± 12.1	53.7 ± 12.0	55.3 ± 13.5
Myocardial mass/BSA (Average, in g/m^2^)	60.1 ± 10.6	56.7 ± 12.2*	55.7 ± 11.4*	58.7 ± 13.5
Peak ejection rate/BSA (ml/s/m^2^)	− 216.4 ± 43.1	− 226.0 ± 36.1	− 227.5 ± 35.6	−233.7 ± 40.8*
Peak filling rate/BSA (ml/s/m^2^)	216.7 ± 26.9	211.6 ± 54.6	211.3 ± 65.1	220.2 ± 41.9
Additional findings				
Late enhancement (*n*)	0	0	0	0
Hepatic steatosis	13	2	0	0

*MRI*, magnetic resonance imaging; *LVEF*, left ventricular ejection fraction; *ED*, end diastolic; *ES*, end systolic; *BSA*, body surface area

*Significant (*P* < 0.05) versus preoperative

### Correlation of Blood Test Results and Cardiac Function

Even though there is a significant decrease of LDL and triglycerides and significant increase of HDL at 12 months after surgery (Table 3), there was no correlation between the improvement of the lipid spectrum and the increase of the LVEF/BSA ratio (*P* = 0.105, *P* = 0.127, *P* = 0.197, and *P* = 0.767 respectively). Additionally, the significant decrease of leptin levels does not seem to influence the LVEF/BSA ratio (*P* = 0.072).

**Table 3:** Blood test results

Variable	Preoperative	Postoperative (months)		
		3	6	12
Lipid spectrum				
Cholesterol	5.2 ± 1.2	4.6 ± 1.1*	4.7 ± 0.9	4.7 ± 0.9
HDL	1.2 ± 0.3	1.2 ± 0.4	1.3 ± 0.4	1.4 ± 0.5*
LDL	3.2 ± 0.8	2.6 ± 1.0*	2.6 ± 0.9*	2.5 ± 0.8*
Triglycerides	2.6 ± 1.4	1.7 ± 0.7*	1.8 ± 1.0*	1.8 ± 1.4*
Metabolic biomarkers				
Leptin	90.6 ± 44.5	33.9 ± 22.0*	26.6 ± 15.0*	18.9 ± 7.7*
Ghrelin	650.5 ± 146.1	691.7 ± 124.3	725.4 ± 260.2	675.6 ± 165.8

*Significant (*P* < 0.05) versus preoperative

## Discussion

This is the first CMRI study to assess cardiac changes after RYGB performed in patients with an uneventful cardiac history. We found a significant increase in LVEF, even after correction for BSA (LVEF/BSA) after RYGB. Additionally, we found a significant decrease in non-corrected cardiac output and absolute LV mass 12 months postoperatively. SV/BSA significantly improved after 12 months and none of the patients showed signs of myocardial ischemia. Due to the necessary increase of cardiac output, needed for enhanced blood supply to the excess peripheral tissue, obesity is associated with a chronic higher cardiac workload as compared to healthy individuals. This will eventually lead to LVH as described by multiple studies [2, 18, 19]. One of these studies was obtained in a group of patients with a BMI > 50, in contrast to our test group with a median BMI of 40.1 preoperatively [20]. As the blood supply to the peripheral tissue decreases after significant weight loss, it is expected that the cardiac workload will change, and therefore the cardiac function will improve after bariatric surgery. In our study, there was no significant change in non-BSA-corrected LVEF after bariatric surgery. Two other studies have found comparable results [21, 22]. It could be possible that in the presence of depressed wall mechanics, ejection fraction is sustained by increased concentricity of LV geometry. A simultaneous reduction of concentricity with improvement in mid wall mechanics is expected to leave ejection fraction unchanged. Nevertheless, due to the significant decrease in BSA, a significant change in LVEF/BSA was seen after 6 and 12 months, despite the small study population. In line with this, there was a significant increase in SV/BSA ratio after 12 months of follow-up. Furthermore, a significant decrease in heart rate and systolic blood pressure was found as a result of loss of volume, and thus a decrease of cardiac afterload. Eventually, there was a significant decrease in cardiac output 12 months postoperatively.

After 3 months, some patients showed a (slight) decrease in EDV and SV. Theoretically, this might be due to lipolysis and/or the cardiodepressive effect of released free fatty acids (FFA) and its associated cardiotoxicity. However, in our population, there was a decrease in serum triglycerides [23]. An explanation for this could be the release of lipid droplets (LDs) which can become cardiotoxic [24].

Besides these theories, it is conceivable that in the first 3 months postoperative, there is a derangement of a stable but adipose state to a katabolic state, which alone has a cardiodepressive effect [25]. The temporary increase in FFA after surgery (due to lipolysis by the acute weight loss) could have a cardiotoxic effect on heart function, just like diabetic cardiomyopathy [26]. Furthermore, there are multiple mechanical changes in cardiac function load and LVF in patients with morbid obesity, for example, increased RV load and OSAS. Because the relative onset of all these cardiac changes is due to the biggest weight loss, the emphasis is on BSA-corrected values. When BSA-corrected values are used, there is an overall improvement as seen in other studies [27].

We detected a 15.2% reduction of LV mass in our patients, which is comparable to other studies measured by CUS [18, 19, 21, 28]. These studies reported mass reductions of 16–22%. However, after correction for changes in BSA, no significant decline was found 12 months postoperatively. There is no evidence that the degree of increase in LVEF and decrease in LV mass is determined by the type of bariatric surgical procedure [29].

In all patients, a significant decrease of BMI (up to 31%) and BSA was found as compared to the preoperative condition, which is to be expected after RYGB. In our study, we have corrected the cardiac function outcomes for BSA as heart function is correlated with BSA and without correction the LVEF would change dramatically. Correction for BSA using the DuBois formula is known for an underestimation of the BSA in patients with obesity of 3% in male patients and 5% in female patients [30]. BSA is generally accepted and widely used to assess cardiac function [31]. The most accurate correction, however, would be with the measurement of the patient’s volume using a 3-dimensional body scanner [32]. Unfortunately, this technique was not available at our hospital during our study.

In a larger study with 312 patients with higher BMI’s, Brownell et al. reported that the presence of LVH was independently associated with BMI ≥ 50 and female sex, after adjusting for age, diabetes, hypertension, and pulmonary hypertension [20]. As there was no significant LVH preoperatively in our group, we cannot confirm this. A possible explanation for the differences in LVH between these test groups and our test group could be the lower BMI in our test group. In one study (*n* = 10), adenosine-induced sub-endocardial ischemia was reported at baseline [9]. Half of the patients in this study underwent bariatric surgery, resulting in complete normalization of ischemia in 3 out of 5 patients and partial improvement in the remaining 2 patients. As determined by CMRI, none of the patients in our study had signs of previous infarction. For logistic reasons, we could not use adenosine CMRI for detection of reversible stress-induced myocardial ischemia.

Hepatic steatosis is closely linked to obesity. This linkage is based on the fact that obesity results in marked enlargement of the intra-abdominal visceral fat depots. The eventual development of insulin resistance leads to continuous lipolysis within these depots, releasing fatty acids into the portal circulation, where they are rapidly translocated to the liver and reassembled into triglycerides [33]. All of our 13 patients had hepatic steatosis preoperatively. This disappeared in 11 patients 3 months after the bariatric procedure. In the other 2 patients, hepatic steatosis decreased significantly and disappeared after 6 months. This all is a direct consequence of diminished intra-abdominal visceral fat depots after RYGB.

There was a significant decrease of LDL and triglyceride levels and increase of HDL 12 months after RYGB. An improved lipid spectrum after RYGB is associated with an improvement of the cardiovascular risk profile [34–36]. However, in our study, no correlation was found between the improvement of the cardiac function and the improvement of the lipid spectrum, which has also been shown in a study in patients with preoperative heart failure [37]. Although cardiovascular risks decline due to an improved lipid spectrum, it does not seem to be related to the improvement of cardiac function. Priester et al. reported that weight loss achieved through bariatric surgery is associated with less coronary calcification and this effect, which appears to be independent of changes in LDL-C, may contribute to lower cardiac mortality in patients with successful gastric bypass [38]. Additionally, Jonker et al. demonstrated that bariatric surgery results in a significant decrease in carotid intima-media thickness in all evaluated age categories, resulting in an improvement of cardiovascular risks [39].

Glucose, leptin, and ghrelin levels could not consequently be measured after fasting due to logistic limitations in CMRI planning (mostly at the end of the day) and patient comorbidity like diabetes.

Therefore, the results of glucose, leptin, and ghrelin outcomes could not be used for analysis. Our study is limited by the small study population. We started with 15 patients, but 2 patients had ustrophobia, even though they had a test visit to the MRI before the study started. The MRI protocol of 45 min was well-tolerated by the other 13 non-claustrophobic patients. Almost all patients stated the breath-holding technique was easier to perform after weight loss. The quality of the CMRI was good and there were no distractions due to the subcutaneous fat. Therefore, we conclude that CMRI is a good technique to assess cardiac function in the population with morbid obesity. Further research in a larger study population is recommended in order to have a better insight of the correlations of different factors in relation to the improvement of cardiac function. It is also recommended to obtain a 3D whole-body scan to measure the whole-body volume for correction of the cardiac function instead of the BSA. In conclusion, this study shows that CMRI is an effective imaging technique to objectively analyze cardiac functional changes in patients with morbid obesity. Also, an improvement of cardiac function after RYGB is seen in patients with morbid obesity without a history of cardiac disease.
